# Marriage Migration Versus Family Reunification: How Does the Marriage and Migration History Affect the Timing of First and Second Childbirth Among Turkish Immigrants in Germany?

**DOI:** 10.1007/s10680-016-9402-4

**Published:** 2016-10-27

**Authors:** Katharina Wolf

**Affiliations:** 1 Population Research Centre, Department of Demography, Faculty of Spacial Sciences, University of Groningen, Landleven 1, 9747 AD Groningen, The Netherlands; 2 Max Planck Institute for Demographic Research, Rostock, Germany

**Keywords:** Fertility, Male fertility, Life-course analysis, Germany, Turkish migrants, Marriage migration, Family reunification

## Abstract

Our study focuses on the fertility of first-generation female and male Turkish migrants in Germany. To evaluate whether timing effects such as fertility disruption or an interrelation of marriage, migration and childbirth occur, we examine first and second births in the years before and after immigration to Germany. The Turkish sample of the Generations and Gender Survey which was conducted in 2006 offers the unique opportunity to examine Turkish immigrants as a single immigrant category. We question the common understanding that Turkish immigrants who arrived to Germany after 1973 mainly arrived for family reunification resulting in high birth intensities immediately after immigration. To distinguish different circumstances under which male and female immigrants have arrived to Germany, we include the combined marriage and migration history of the couple. We find that first birth probabilities are elevated during the years immediately following migration. But this effect is not universal among migrants with different marriage and migration histories. It appears that the arrival effect of high birth intensities is particularly high among female immigrants and is evident only among marriage migrants, that is Turks who married a partner who already lived in Germany at the time of the wedding. By contrast, among those who immigrated for family reunification, we do not find such an arrival effect.

## Introduction

Since the 1950s, net migration of foreigners to Germany has, on average, been positive (Statistisches Bundesamt 2014). As a result, the number of people of foreign origin has been growing since that time (Statistisches Bundesamt 2013). Of this population, individuals of Turkish origin form the largest group, making up 3.6 % of the total population residing in Germany in 2011 (Statistisches Bundesamt 2012). Since migration is an incisive event in the human life course, it can be expected to have a strong impact on the occurrence and timing of childbirth. In particular, fertility outcomes depend to a large extent on the timing of migration over the life course (Adserà and Ferrer 2014). Thus, migrant fertility must be explored from an individual life-course perspective. We investigate each migrant’s full fertility history by examining the births of his or her children which occurred before migration (most likely in Turkey) and after migration (in Germany). For the first time, not just female, but also male immigrant fertility is under study. Furthermore, instead of focusing on comparing migrant fertility with the fertility of Germans, this study is among the few that focuses in detail on migrant fertility only (see also Schmid and Kohls 2009; Stichnoth and Yeter 2013). We follow this approach because it allows us to evaluate the impact of a number of migration-specific indicators. Recent studies have shown the importance of the duration of stay in the host country. For example, birth intensities were found to be high during the time immediately following migration among several migrant groups in Europe and the USA (Carter 2000; Lübke 2014; Milewski 2007; Toulemon 2004). In addition, the age at migration and the reasons for migrating have been shown to have affected migrant fertility (Andersson 2004; Cygan-Rehm 2011; Milewski 2007; Mussino and Strozza 2012; Toulemon 2004). In the case of Germany, researchers have stated that high birth intensities immediately after immigration might be related to the fact that most of the migrant women in Germany arrived for family reunification (e.g. Milewski 2007). However, to our knowledge, in none of the previous papers on that topic migration for family reunification and other forms such as marriage migration have been distinguished sufficiently. To shed more light on different pathways of entering Germany and the effects on migrants’ fertility behaviour, we take into account the combined marriage and migration history of the couple and compare immigrants arriving for family reunification to those who come as marriage migrants.

Our research questions are as follows: What is the relationship between first and second childbirth and the duration of stay in Germany among male and female Turkish immigrants? Are birth risks highest in the years preceding migration, in the years immediately after the move, or in the years that follow? And, how do fertility patterns differ by age at migration and are there differences between marriage migrants and those who migrated for family reunification? As our data source, we use the first wave of the German Generations and Gender Survey (GGS), which was conducted in 2006. The immigrant sample includes Turkish citizens of the first migrant generation who were living in Germany. In a first step, the age-specific fertility rates, along with the total fertility rates, are estimated by age at migration. This provides us with an initial impression of the differences in fertility between male and female Turkish migrants. In our multivariate analysis, we use discrete-time regression models to examine the risk of having a first and a second birth by duration of stay separately for men and women. We furthermore investigate the impact of the age at migration and compare marriage migrants with those who migrated for family reunification.

## Theoretical Considerations and Previous Research

### Turkish Migration to Germany

Coordinated labour migration from Turkey to Germany began in 1961 and ended in 1973. After the recruitment agreement was halted, there were only few possibilities to immigrate legally from Turkey to Germany (Münz et al. 1999; Seifert 1997). Turkish immigrants could either rely on their right of asylum or migrate under the family reunification law (“Familienzusammenführung”). The latter allows an immigrant’s foreign spouse and children below age 16 to immigrate. There are two major categories of Turkish immigrants arriving under the family reunification law, namely those who were married before one of the partners migrated to Germany and who arrived to reunify with their spouse and, second, those who married a spouse already living in Germany and then immigrated to Germany to join their spouse. The latter are referred to as transnational marriages which were and still are quite common. Almost half of all married first-generation Turkish migrant men living in Germany married a wife who was living in Turkey at the time of the wedding. But the share of transnational marriages is smaller among Turkish women (Kalter and Schroedter 2010). The preference for transnational marriages among Turkish immigrants in Germany also diminishes across generations: Among second-generation Turkish immigrants, a second-generation Turkish partner is the dominant choice (Huschek et al. 2012; Kalter and Schroedter 2010). While the practice of transnational marriage is related with low educational levels among men of Turkish origin in Germany, this is not the case for women (González-Ferrer 2006).

In recent decades, Turkey has experienced a steep fertility decline. The TFR fell from more than six children per woman in 1950 to 2.07 in 2013. Over the same period, the decline in the TFR was accompanied by an increase in the mean age at childbirth, from 26.7 to 27.7 years (Statistics Turkey 2014; United Nations Population Division 2012). We can therefore assume that the decrease in the total fertility rate was partly driven by a postponement of childbirth to higher ages. Recent parity-specific analyses have shown that the level of childlessness in Turkey is still low, but the risks of having a third or fourth child have declined sharply since the 1990s (Yavuz 2008). Fertility levels in Turkey also differ considerably between rural and urban regions. For example, in 2003, the TFR of women living in urban environments was about 1.68, whereas the TFR of women living in rural areas was, at 3.63, more than twice as high (Eryurt and Koç 2012). While the development of fertility patterns in Turkey should not be ignored in studies of Turkish migrant fertility, there is, unfortunately, no simple way to take these trends into account. In our multivariate regression models, we include a migrant’s birth cohort to control for changes across time. Nevertheless, we do not know to what extent migrants have been influenced by the development of fertility in Turkey. Most migrants in our sample left the country when they were young adults. The degree to which they are still influenced by family and fertility values in Turkey depends not only on their level of integration and their social environment in Germany, but also on the number and the intensity of their contacts in Turkey. Since we do not have any information on those indicators, we should be careful when interpreting our results on Turkish migrant fertility, and bear in mind that we cannot draw any conclusions regarding the influence of value shifts which have been taking place in Turkey.

The development of fertility in Turkey, in combination with the history of Turkish migration to Germany, has several implications for our study. Over the past century, Turkey has had a lower prevalence of childlessness and higher fertility levels than western European countries. The sharp decline in fertility was mainly driven by lower birth intensities of higher birth orders. Since Turkish immigrants in Germany were born in Turkey, and were, at least partly, socialized in a high fertility context, we expect to find that those first-generation immigrants had low levels of childlessness and high first and second birth intensities. This assumption is further supported by the fact that the majority of Turkish immigrants in Germany are from rural areas in Turkey, where fertility levels continue to be higher than they are in urban regions. Our sample of Turkish immigrants mainly consists of men and women arriving in Germany after 1973. Thus, our focus is on the two major immigrant groups arriving after that time, that is, migrants who arrived for family reunification and marriage migrants. We therefore take into account at which point in time the couple has married: before or after both partners migrated to Germany or after only one of the partners migrated.

### Fertility Disruption, the Interrelation of Events, and the Selectivity of Immigrants

Four major “partly complementary, partly contradictory hypotheses” (Kulu 2005, p. 52) have been advanced by demographic researchers to explain migrant fertility. Scholars have variously attributed migrants’ fertility behaviour to disruption, selection, socialization, and adaptation effects (Hervitz 1985; Kulu 2005; Lee 1992; Rundquist and Brown 1989; Singley and Landale 1998; Stephen and Bean 1992). In addition to these theoretical approaches, empirical evidence of an interrelation of events has been suggested (Andersson 2004; Milewski 2007). Socialization and adaptation arguments are of minor relevance for this paper, as they are based on comparisons of migrants and the majority population in the country of destination (Alders 2000; Andersson 2004; Hervitz 1985; Kahn 1988; Lindstrom and Saucedo 2002; Singley and Landale 1998; Stephen and Bean 1992). By contrast, analyses which investigate disruption and the interrelation of events focus on migration and childbirth timing without focusing on a comparison with the majority group population. Selection effects are also highly relevant for the study of migrant behaviour. Arguments based on disruption, the interrelation of events, and selection are therefore discussed below.

#### Fertility Disruption

According to disruption theory, the economic and the psychological costs of migration cause stress, which in turn leads to temporary discontinuities in childbearing behaviour. Disruption might occur in the years immediately prior to migration, during the process itself, and shortly after arrival at the destination (Goldstein 1973; Hervitz 1985; Kulu 2005; Stephen and Bean 1992). The so-called anticipatory effect is based on the assumption that low childbirth intensities immediately prior to migration may be caused by stress related with the organizational planning of the move, a temporary separation from the partner, or economic hardship. In the years immediately following migration, conception is considered unlikely because individuals need some time to settle in, and to find proper housing and employment. Empirical evidence of temporary fertility disruption was found for several countries and migrant populations. Perez-Patron (2012) found indicators of post-migration disruption among Mexican migrants in the USA, if migration occurred prior to the start of family formation. In addition, ethnic Germans (“Aussiedler”) have been shown to have experienced disruption in the period immediately after they arrived in Germany (Dinkel and Lebok 1997). Other studies found that fertility was disrupted prior to migration among Mexican immigrants in the USA and among several immigrant groups in Germany, Italy, Canada, and Spain (Carter 2000; Choi 2014; Milewski 2007; Mussino and Strozza 2012; Ng and Nault 1997; Vila and Martìn 2007). For Turkish migrants in Germany, we expect to find pre-migration disruption, namely low first and second childbirth risks, among marriage migrants but not among family reunifiers (H1a). This is due to the fact that, for most of the marriage migrants, the migration to Germany is one of the last steps in the family formation process. Typically, partners get engaged while one of the partners lives in Germany, but the other one still lives in Turkey. The engagement festivities are followed by a period of partners' separation that lasts until the partner finally follows his or her partner and migrates to Germany (Aybek 2015). Family reunifiers also experience periods of separation of the spouses, but the couples are already married and may visit each other. Post-migration disruption indicated by low first and second birth intensities during the years shortly after immigration may occur among both marriage migrants and family reunifiers, because both groups need time to settle in and thus might postpone fertility (H1b).

#### Migration and the Interrelation of Events

Migration and birth decisions are important life-course decisions which must be studied from a life-course perspective (Kley 2011; Willekens 1991; Wingens et al. 2011). It is generally understood that the process of migration is strongly associated with family formation events such as marriage (Mulder and Wagner 1993). On the one hand, changes in family life, such as union formation or childbirth, strongly determine migration decisions. On the other hand, migration has an important influence on the timing of family-related events. It is therefore assumed that the elevated birth rates observed among migrants shortly after arrival result from an interrelation of migration, union formation, and childbirth (Andersson 2004; Singley and Landale 1998). A large body of research has shown that there is a close link between family formation and migration, i.e. that fertility is particularly high immediately after migration. This link has been demonstrated for immigrants in the USA, France, Sweden, Spain, the UK, and the Netherlands (Alders 2000; Andersson 2004; Andersson and Scott 2005; Carter 2000; Choi 2014; Devolder and Bueno 2011; Lindstrom and Giorguli-Saucedo 2007; Lübke 2014; Singley and Landale 1998; Toulemon 2004). Studies on Spain and Italy showed that period fertility and first birth risks were particularly high shortly after arrival among women who migrated for family reasons (Mussino and Strozza 2012; Vila and Martìn 2007). In Germany, the first birth risks of guest worker immigrants from Turkey, Italy, Spain, Greece, and former Yugoslavia were found to be elevated in the first year after immigration and were particularly high in the first year of marriage (Milewski 2007). Based on the empirical findings on the close relationship between migration, marriage and the transition to parenthood, we expect marriage migrants from Turkey to have high first birth risks shortly after they have arrived in Germany (H2). Obviously, this contradicts hypothesis 1b on post-migration disruption.

One of the major contributions of this paper is the distinction between male and female Turkish immigrant fertility patterns. We expect to find gender differences particularly among marriage migrants. Generally, marrying a co-national partner, who was still living in the country of origin at the time of the marriage, has been found to be related with a migrant’s strong orientation towards the traditions, norms, and values that are dominant in the home country. But scholars are divided over the question whether this applies to male and female migrants in the same way (see, e.g. Baykara-Krumme and Fuß 2011; González-Ferrer 2006). Some argue that migrant women who marry a partner from Turkey orient themselves less towards the traditional family role model and tend to marry a partner from the home country as part of an emancipatory process, because such a setting offers a larger autonomy from the family-in-law (Lievens 1999; Timmerman et al. 2009). In addition, qualitative research reveals that the sequence of events such as getting engaged, moving to Germany, and celebrating the wedding party differs among male and female marriage migrants. It appears that women who marry a man from Turkey hold positions of power that allow them to organize the wedding and migration of the partner according to their own preferences (Aybek 2015). Van Landschoot et al. (2014) show that partner choice patterns of the Turkish second generation in Belgium have a clear impact on their fertility patterns. First birth risks are higher among Turkish second-generation women with a first- or second-generation partner from Turkey, compared to those with a native Belgium partner. The lack of a significant difference in first birth risks between those who are partnered with a first or a second-generation Turkish migrant may be interpreted as an indicator of the instrumentality of marrying a first-generation partner for emancipatory reasons (Van Landschoot et al. 2014). For first-generation Turkish migrants in Germany we state the following hypotheses. If men living in Germany choose a partner from Turkey to maintain the traditional family role model, then their wives who follow would show high first and second birth intensities after immigrating to Germany (H3a). If women living in Germany marry a partner from Turkey to emancipate themselves from the traditional family model, we would expect lower first and second childbirth risks in those cases where the men follow (H3b).

#### Selectivity of Migration

While classical selection theory is most often used to explain differences between migrants and non-migrants in the destination country (Goldstein and Goldstein 1981; Ribe and Schultz 1980), it also provides a framework for comparing migrants to non-migrants in the country of origin. Several scholars have attributed the high levels of immigrant fertility to the fact that immigrants are positively selected in terms of fertility relative to the stayers in the country of origin (Choi 2014; Dubuc 2012; Frank and Heuveline 2005). By comparing migrant populations in Italy and Russia, Mussino and Van Raalte (2013) concluded that immigrants tend to have similar first birth risk profiles, even though they originate from and migrated to different countries. This suggests that migrants are a selective group who display behaviour which might be determined less by country-specific circumstances than by the fact of being a migrant (Mussino and Van Raalte 2013). In addition, recent research has extended the conventional notion of selection theory, positing that selectivity might also occur in terms of the reasons for migration and the individual’s or the couple’s life stage (Kulu and González-Ferrer 2014, p. 422). According to Lindstrom and Saucedo (2002), the selectivity of migrants depends on whether migration is temporary or permanent. Migration streams also appear to become less selective over time (Frank and Heuveline 2005; Portes 1979). Chain migrants are thus less selective than the group of pioneer migrants who moved before a large migration stream had developed (Massey 1990). We assume that marriage migrants and those who arrive for family reunification have a strong family orientation, which is accompanied by a selectivity towards high fertility intentions. However, as mentioned before, male marriage migrants may form an exception as it has been found that Turkish women in Germany often choose to marry a partner from Turkey to emancipate themselves from the family-in-law and the traditional family model. As a result, the men who arrive as marriage migrants would also have more modern views on family and childbirth. Based on this gender difference in selectivity and in line with hypotheses 3a and 3b, we expect that male marriage migrants show lower first and second childbirth intensities than female marriage migrants.

## Data and Methods

### Data and Sample

Our main data source is the first wave of the German Generations and Gender Survey (GGS). It includes a sub-sample of Turkish first-generation immigrants who were drawn from all Turkish citizens aged 18–79 who were registered in Germany in 2006 (*n* = 4000). This implies that the Turkish-born who hold German citizenship are under-represented, but the share of Turkish immigrants in Germany who naturalized was only 21 % in 2005 (Bandorski et al. 2007). Although the GGS is a cross-sectional dataset and the data were collected in the receiving country only, detailed birth histories and a number of migration-specific covariates are provided. As a result, the date of immigration and the current partner’s migration history are available for our analysis. The interviews were held in German, but the questionnaires were also available in Turkish. The same applies to the information brochures for the first contact between interviewers and respondents. Therefore, selection towards those with good German language skills should be minimized. We restrict our sample to women and men born between 1950 and 1969, because the birth histories in the GGS sample were found to be biased for respondents born before 1950 (see Kreyenfeld et al. 2010; Sauer et al. 2012), and only respondents who had already experienced most of their fertile life span were to be included. Even though we are examining both male and female fertility behaviour, we do not take a couples perspective. The men and women in our sample are independent individuals. The final sample consists of men and women of the birth cohorts 1950–1969. Thus, the respondents were aged 36–55 at the time of the interview. We also excluded respondents with missing information on their age, their date of immigration, or their birth history. Only the biological children of the respondents were taken into account. As shown in Table 1, our final sample size consists of 1125 respondents who were at risk of having a first child, of whom 550 are male and 575 are female. As Table 5 in the Appendix illustrates, the sample for second births is slightly smaller (*n* = 1050).

**Table 1:** Number of occurrences and exposures of first birth

	Women		Men	
	Person months at risk (%)	Number of first births	Person months at risk (%)	Number of first births
Birth cohort				
1950–1954	19.3	97	11.0	45
1955–1959	19.3	102	19.7	96
1960–1964	26.1	143	30.8	165
1965–1969	35.3	203	38.5	199
Education				
Low	72.4	406	47.4	248
Intermediate	14.4	76	33.5	173
High	5.5	17	11.8	46
Other	7.7	46	7.4	38
Age at migration				
0–14	26.9	152	34.8	184
15–19	18.8	132	17.8	109
20–24	25.1	141	18.6	100
25–29	10.6	53	15.1	61
30+	18.7	67	13.7	51
Marriage and migration history				
Family reunification				
Marriage, R migrated, partner migrated later	3.1	17	4.1	19
Marriage, partner migrated, R migrated later	5.7	42	0.6	3
Marriage migration				
R migrated, marriage + partner migrated	11.7	64	29.7	173
Partner migrated, marriage + R migrated	19.5	127	9.2	51
Other groups				
Marriage before/at joint couple’s migration	8.6	51	5.8	34
R and partner migrated, married later	16.5	106	19.5	115
Never married	3.3	15	7.0	23
Missing info on marriage or partner’s migration date	31.5	123	24.2	87
No. of respondents at risk		575		550
No. of birth events		545		505

*Notes*: R stands for respondent, a comma means that the event took place after the previous event whereas + means that both events took place around the same time. *Data*: German GGS 2006, unweighted

### Methods

Following the approach by Toulemon (2004) (see also Devolder and Bueno 2011; Toulemon and Mazuy 2004), we estimate age-specific fertility rates in order to gain a first impression of the fertility patterns of Turkish migrants in Germany. The rates fluctuate considerably given the small sample size. The curves are smoothed using a 3-year moving average. The total fertility rates (TFR) are then calculated. TFRs should be used with caution when studying immigrant populations, as these rates may be biased by tempo effects, as well as by other factors specific to immigrants, such as the age at migration or the marital composition (Parrado 2011). One of the basic assumptions is that the age groups are homogeneous, which is not the case for migrants, as their fertility differs by migration stage. Hence, we group Turkish fertility rates by age at migration to examine whether different patterns evolve from varying life-course experiences. To learn more about the impact of the age at migration on childbirth, we model the individual life courses with the help of event plots. These plots are a useful tool to evaluate the interplay of several life-course events graphically (Willekens 2014). Each line corresponds with one individual in our data. For each person, the age at migration is labelled with a circle and a cross stands for the birth of the first child. In addition, we split the group of migrants by the marriage and migration history of the couple. These graphic presentations allow us to gain some insight into the associations in the timing of events. This can be particularly useful when the sample sizes are small, and the statistical power of regression models is therefore limited.

For the multivariate analysis, we estimate discrete-time regression models which are based on a logistic link function. $P_{it}$ denotes the probability of having a child at year *t* for individual *i*. The term $\alpha _0$ describes the baseline hazard, and $\beta ^{\prime}$ represents the estimated regression coefficients for covariates *x*. The simple regression model is expressed by Eq. (1):

1 $$\ln \left( {\frac{P_{it}}{1-P_{it}}}\right)= \alpha _{0}(t) + \beta ^{\prime } x_i(t) + \beta ^{\prime } x_i$$

### Covariates

Our model contains time-varying ($x_i(t)$) as well as time-constant covariates ($x_i$). The time-varying covariate is the number of years of duration of stay in Germany *d*. It has negative values if the child was born before the parent migrated, and positive values if the childbirth occurred after the migrant’s arrival in Germany. The duration of stay *d* is grouped into the following categories: *d* ≤ −3 years, −3 < *d* ≤ −1, −1 < *d* ≤ 0, 0 < *d* ≤ 1, 1 < *d* ≤ 3, 3 < *d* ≤ 6, 6 < *d* ≤ 9 and 9 < *d* years. The duration of stay always relates to the immigration of the individual, irrespective of the partner’s migration timing. When dealing with negative durations, the results should be regarded with caution. Hoem (2014) illustrated how easily people draw false conclusions when migrant fertility is examined only on the basis of data on those who actually migrated (for different examples, see also Hoem and Kreyenfeld 2006a, b). Since birth intensities vary with migration intensities, the group of individuals who migrated differ in terms of their fertility from the stayers in the country of origin.

As time-constant covariates, we include the couple’s marriage and migration history, the birth cohort, the educational status and the age at immigration of the respondent. Since the migrants who arrived in childhood were not yet in their reproductive phase, they were not at risk of changing their behaviour in response to migration. For that reason, our sample for the multivariate analysis is restricted to women and men who arrived in Germany after their 15th birthday. As both the covariate of the current age and the age at migration are highly correlated with the duration of stay and with each other, we could not include both age covariates in our models at the same time. Since the age at migration is of major importance for migrant fertility patterns, we decided to make use of this covariate. The results of the regression model using the current age instead do not differ and are available from the author upon request. Information on education is based on the ISCED code and was grouped into the following categories: *low education* (ISCED code 1–2: primary or lower secondary school degree), *intermediate education* (ISCED code 3: upper secondary school degree), *high education* (ISCED code 4–6: post-secondary or tertiary degree), and *other education* (ISCED code 7: still in school or in training, other educational degree, unknown status).^1^ Respondents were grouped into the following birth cohorts: 1950–1954, 1955–1959, 1960–1964, and 1965–1969. To include the partners’ marriage and migration history, we evaluate the time of their wedding (relative to the respondent’s and the partner’s immigration date) and consider which of the partners migrated to Germany first and who followed later on. According to these indicators, the Turkish migrant population in Germany was divided into three categories: those coming for family reunification, marriage migrants and others. Family reunifiers are respondents who had married before one of the partners migrated to Germany and reunified later on. They are further distinguished into two sub-categories: migrants who came to Germany first (whereas their partners followed later) and migrants who followed their partners. Our second category is marriage migrants, who married after one of the partners had already migrated to Germany, while the other spouse followed after marrying. We also make the distinction between first movers and followers. A third group of people consists of immigrants with other migration and marriage histories. It contains those who migrated at the same time as their partner, those who have never been married and respondents who married after both partners have migrated to Germany. The latter category might include respondents who migrated on their own account and met each other later, but it could also contain marriage migrants who followed a partner and married after arrival in Germany. To further distinguish these subgroups, we would need more detailed information about the distance in time between the arrival of the partners and the wedding, but our sample is too small to do so. A last category contains all cases where no information on the combined marital and migration history was available. This is necessary because, unfortunately, the GGS data offer information on the partner’s migration date only for the respondent’s current partner at the time of the interview. However, the share of respondents who were with their current partner (at the time of the interview) even before they migrated to Germany is with 68.5 % among women and 75.8 % among men quite high.^2^ For the regression models on second births, the duration since the first birth is included as well. We distinguish between births which occurred in the year after the previous birth, and those which occurred in the second, the third, or a subsequent year. In addition, we include a dummy variable indicating whether the first child was born in Germany.

## Results

### Descriptives

**Fig. 1:**
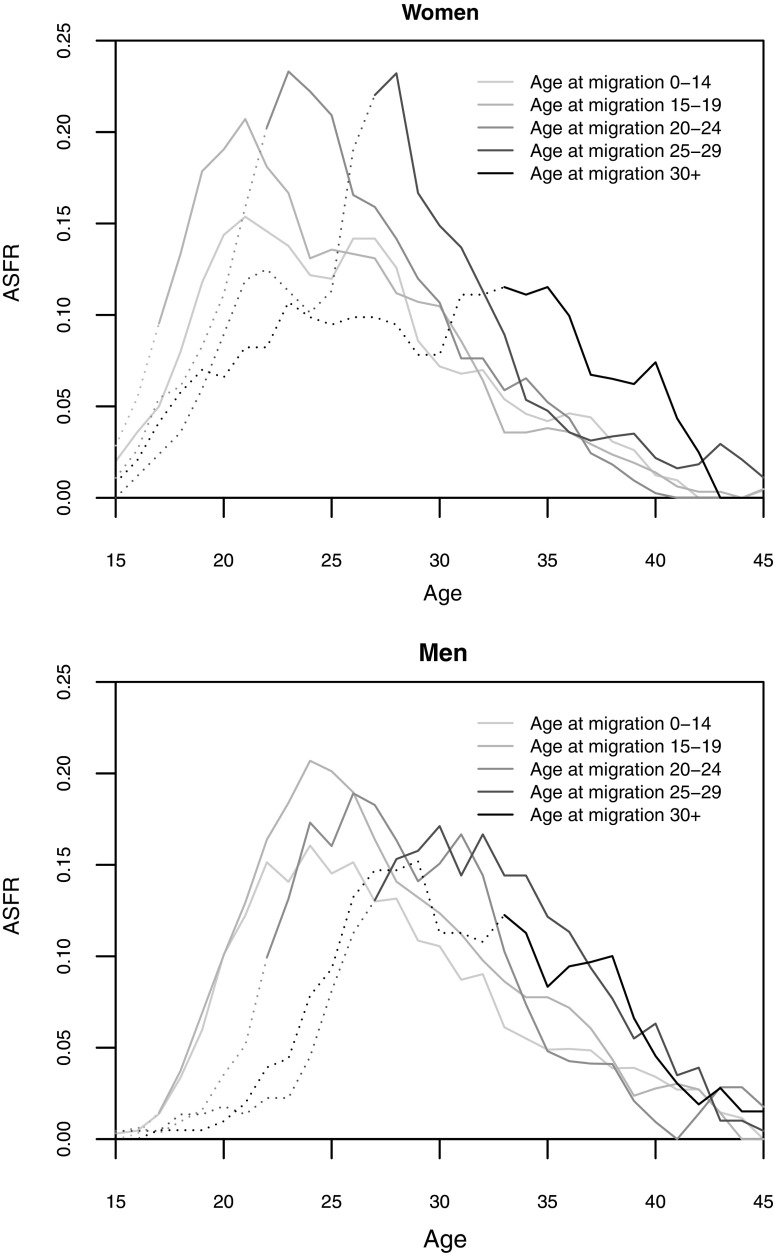
Age-specific fertility rates by age at migration, three-year moving averages. *Notes*: The *dotted lines * mark the time before migration, the *solid lines* the time after migration. *Data*: German GGS 2006, unweighted

Figure 1 on page 12 shows the age-specific fertility rates by age at migration, separately for male and female Turkish immigrants in Germany. We find that the age-specific fertility rates were highest in the years following migration among those who migrated after age 14. These peaks seem to be more pronounced among women. The effect is slightly postponed among men. Immigrants who arrived before age 15 or after age 29 showed the lowest age-specific fertility rates. However, the pattern among the female immigrants who arrived in Germany at age 30 or older seems a bit odd. This is, however,  less surprising considering that the group of respondents on whom this result is based is very small. Unfortunately, due to the small sample size, it was not possible to use separate categories for migrants arriving between age 31 and 40 and those who came after age 40. As a result, our category “30 and older” contains only a few respondents who migrated after their childbearing ages. The total fertility rates grouped by age at migration confirm our findings on high fertility among those who arrived in young adulthood. Table 2 shows that migrants who arrived in Germany before age 15 or after age 29 have had significantly lower TFRs than those who arrived in young adulthood. Among Turkish men, the mean age at first childbirth (MAC_1_) was higher the older the migrant was at migration. Turkish women who moved to Germany before age 19 also showed an elevated MAC_1_. As it appears there is a strong relationship between the age at migration and the fertility patterns of the Turkish migrants in Germany, this heterogeneous group should be analysed separately by age at migration.

**Table 2:** Total fertility rates and mean ages at first childbirth (and 95 % confidence intervals) by age at migration

Age at migration	TFR (95 % CI)		MAC_1_ (95 % CI)	
	Male	Female	Male	Female
0–14	2.08 (2.02, 2.14)	2.15 (2.07, 2.23)	24.4 (23.6, 25.2)	25.2 (25.1, 25.3)
15–19	2.61 (2.47, 2.75)	2.49 (2.44, 2.54)	24.3 (23.5, 25.1)	26.0 (25.8, 26.2)
20–24	2.29 (2.27, 2.31)	2.49 (2.44, 2.54)	26.2 (26.1, 26.3)	23.0 (22.0, 24.0)
25–29	2.19 (2.17, 2.21)	2.44 (2.40, 2.48)	28.4 (27.6, 29.2)	24.2 (23.7, 24.7)
30+	2.04 (1.96, 2.12)	2.18 (2.11, 2.25)	28.9 (27.9, 29.8)	28.9 (27.6, 30.3)
All Turks	2.28	2.35	25.7	24.9

*Data*: German GGS 2006, unweighted

In the event plots in Fig. 3 (in the Appendix), each line corresponds with one respondent in our sample. The first panel shows marriage migrants who married after one of the partners had moved. It seems that for this group, first childbirth occurs mainly after the respondent has migrated to Germany. Particularly for those who arrived between the ages of 20 and 30 childbirth seems to happen quite often immediately after arrival. The second panel illustrates the event plot for respondents who were already married before migration and who reunified in Germany later on. It appears that in those cases, many respondents had their first child before migration. The findings based on the event plots indicate that there is no post-migration disruption as has been suggested in hypothesis 1b, neither among marriage migrants nor among family reunifiers. Instead, the pattern among Turkish marriage migrants is in line with our second hypothesis, that is based on a short time interval between marriage, migration and family formation, marriage migrants tend to have their first child immediately after immigration to Germany.

### Multivariate Analysis

#### First Birth

Table 3 provides the results of the regression model on first births among female and male Turkish migrants based on average marginal effects (AME). We find a strong effect of the duration of stay in Germany. The probability to have a first child is lowest in the years preceding migration, highest within the first year after migration, and decreases in the following years. Furthermore, first birth probabilities are highest among women who arrived in young adulthood between the ages of 15 and 24, and lowest among those who came after age 30. In addition, we do not find any significant effect of the birth cohort of the women or of their educational attainment. Further analyses revealed that the absence of a cohort effect is not due to a correlation between age at migration and cohort. The impact of the combined marriage and migration history of the couple is also rather small and not significant. However, it seems that female reunifiers, namely those who have been married before both partners’ migration, have lower probabilities of having a first child compared with marriage migrants. Among Turkish migrant men, the only covariate that has a significant impact on first birth probabilities is the duration of stay. As for females, first birth intensities are low before migration and peak in the year immediately following migration. Even though the age at migration is an important determinant of first birth behaviour among female migrants, there is no significant effect for males. The coefficients of the covariate on the marriage and migration history show that respondents involved in marriage migration have the highest probabilities of having a first child, but there is no significant difference compared with the other groups. Our findings indicate that there is a disruptive effect on first birth intensities of men and women in the years preceding migration. Whether this applies to the same extent to subgroups with different marriage and migration histories is examined in the next step.

**Table 3:** Average marginal effects (AME) on first birth among female and male Turkish migrants

	Women		Men	
	AME	*P* value	AME	*P* value
Duration since migration (years)				
*d* ≤−3	−0.045	0.098	−0.125	0.000
−3 < *d* ≤ −1	−0.106	0.000	−0.130	0.000
−1 < *d* ≤ 0	−0.087	0.001	−0.105	0.000
0 < *d* ≤ 1	Ref.	–	Ref.	–
1< *d* ≤ 3	−0.056	0.016	−0.046	0.134
3< *d* ≤ 6	−0.144	0.000	−0.103	0.000
6< *d* ≤ 9	−0.160	0.000	−0.080	0.002
9 < *d*	−0.218	0.000	−0.185	0.000
Birth cohort				
1950–1954	0.000	0.994	0.005	0.881
1955–1959	Ref.	–	Ref.	–
1960–1964	−0.003	0.899	−0.017	0.489
1965–1969	−0.025	0.255	−0.020	0.938
Age at migration				
15–19	Ref.	–	Ref.	–
20–24	−0.046	0.022	−0.020	0.418
25–29	−0.073	0.001	−0.029	0.266
30+	−0.121	0.000	−0.025	0.416
Education				
Low	Ref.	–	Ref.	–
Intermediate	0.008	0.798	0.001	0.946
High	−0.001	0.985	−0.026	0.377
Other	−0.010	0.708	−0.005	0.854
Marriage and migration history				
Family reunification				
Marriage, R migrated, partner migrated later	−0.005	0.891	−0.018	0.641
Marriage, partner migrated, R migrated later	−0.024	0.382	0.009	0.923
Marriage migration				
R migrated, marriage + partner migrated	0.065	0.161	0.012	0.701
Partner migrated, marriage + R migrated	Ref.	–	Ref.	–
Other groups				
Marriage before/at joint couple’s migration	−0.018	0.476	−0.009	0.779
R and partner migrated, married later	0.049	0.158	0.013	0.681
Never married	−0.005	0.906	−0.008	0.852
Missing info on marriage or partner’s migration date	0.025	0.324	−0.009	0.762
No. of birth events		545		505
AIC		1898.6		1692.6

*Notes*: R stands for respondent, a comma means that the event took place after the previous event whereas + means that both events took place around the same time.

*Data*: German GGS 2006, unweighted

**Fig. 2:**
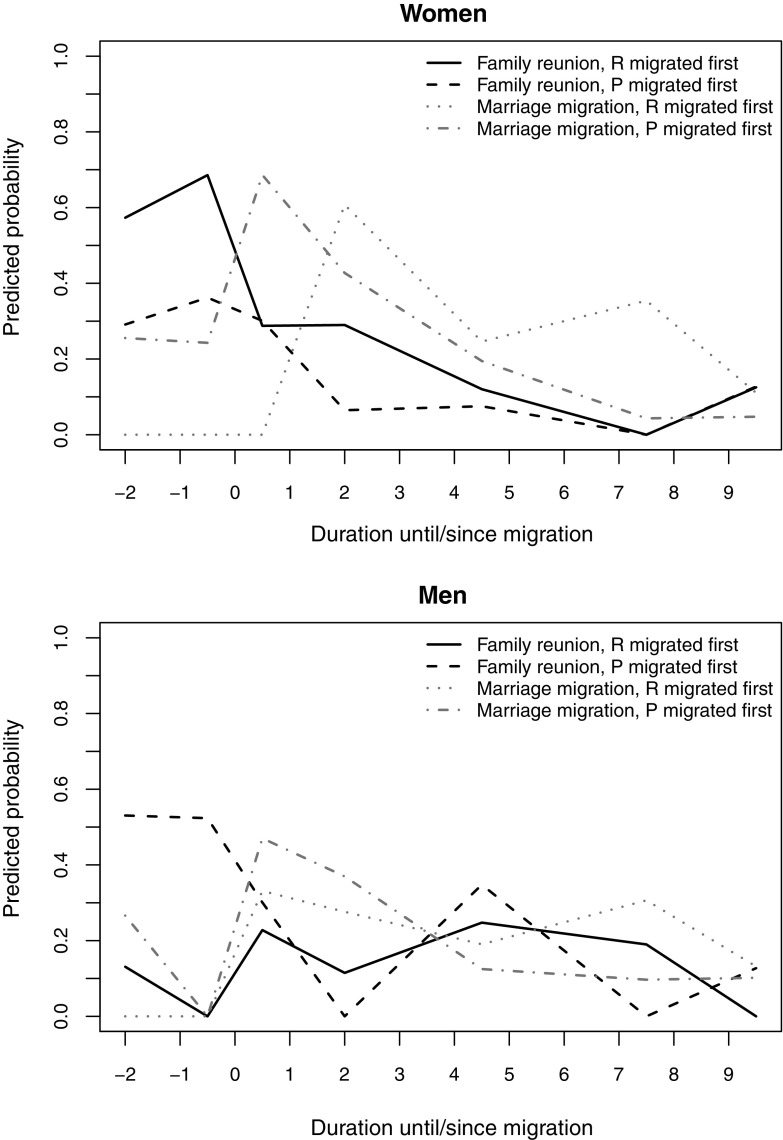
Predicted probabilities of having a first child by duration of stay and marital/migration history. *Notes*: Controlled for birth cohort, age at migration, education. *Data*: German GGS 2006, unweighted

**Fig. 3:**
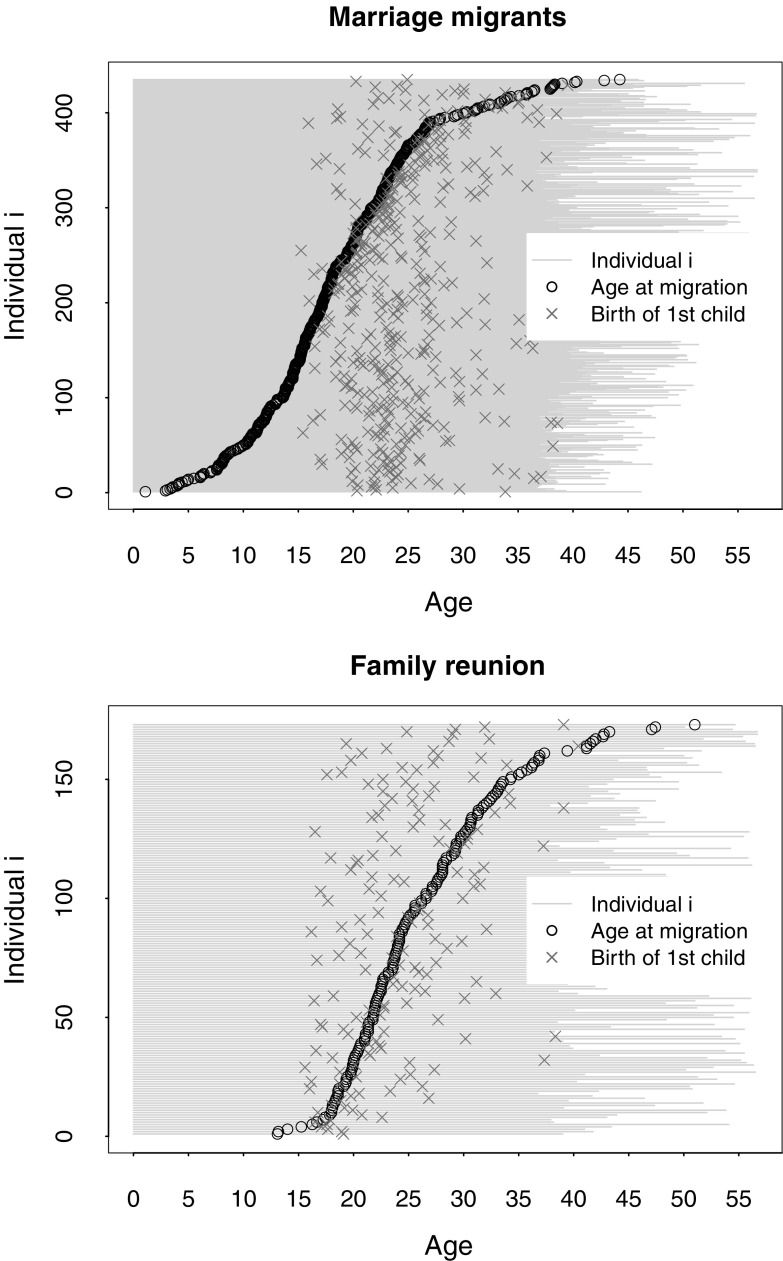
Event plot, occurrences of first births by age at migration among migrants who arrived after age 14, separated by marriage and migration history of the couple.* Data*: German GGS 2006, unweighted

Since we are particularly interested in investigating male-female differences in childbirth patterns and compare those among family reunifiers and marriage migrants, we estimated an interaction effect with the duration of stay. The resulting predicted probabilities are shown in Fig. 2 (Table 6 in the Appendix lists the average marginal effects). The graphs include a selection of migrant groups, namely those respondents married before their own or the partner’s migration (family reunification) and those who married after one of the partners migrated to Germany (marriage migration). We do not find any evidence for low first birth intensities among family reunifiers, but first birth risks are low among marriage migrants in the years preceding migration. Thus, again in line with our first hypothesis, pre-migration disruption seems to occur only among marriage migrants. Respondents involved in family reunification, who were married before both partners migrated, show higher probabilities of having a first child in the years before migration and low probabilities afterwards. Thus, as expected, migration, marriage and first childbirth seem to be highly interrelated among Turkish marriage migrants, but not among family reunifiers (H2). Among female marriage migrants, we find high first birth intensities in the years following migration. A similar effect but shifted by 2 years appears among women who married a marriage migrant from Turkey. The second panel in Fig. 2 shows the results for our male sample. It appears that there is an arrival effect of high first birth probabilities among male marriage migrants, but it seems to be slightly less pronounced than in the female sample. We find that female marriage migrants from Turkey show high first birth risks after immigration, as suggested by our gender-related hypothesis 3a. Among male marriage migrants, however, we did not find any evidence for low first birth risks after immigration that would reflect more modern family values (H3b).

#### Second Birth

The results on second births are shown in Table 4 for women and men. In our model, for females, the duration of stay again has an important effect on the probability of having a second child. Contrary to the disruption hypothesis, second birth probabilities are high in the years before migration, again peak in the year of immigration, and decreases significantly in the following years. The effect of the covariate on the duration since the first childbirth indicates that the probability of having a second child was highest in the second year after the first childbirth. In addition, it seems to matter whether the first child was born before or after migration. The probability of having a second child was higher among respondents whose first child was born in Germany than among those whose first child was born in Turkey. We did not find any significant differences by birth cohort, educational level, age at migration, or the marriage and migration history of the couple. Our model for men indicates that only the duration since first birth and whether the child was born in Turkey have a significant impact on second birth risks. Those are highest in the second and third year after the birth of the first child. As for females, the probability of having a second child increases, if the first child was born after migration to Germany. The probability of having a second child does not vary significantly by duration of stay in Germany. Unfortunately, the number of cases in each category is too small to estimate the interaction effect between the duration of stay and the combined marriage and migration history of the couple for second births.

**Table 4:** Average marginal effects on second birth among female and male Turkish migrants

	Women		Men	
	AME	*P* value	AME	*P* value
Duration since migration				
*d* ≤ −3	−0.097	0.095	0.113	0.485
−3 < *d* ≤ −1	−0.128	0.020	0.059	0.736
−1 < *d* ≤ 0	−0.090	0.243	−0.029	0.872
0 < *d* ≤ 1	Ref.	–	Ref.	–
1 < *d* ≤ 3	−0.173	0.000	−0.047	0.720
3 < *d* ≤ 6	−0.258	0.000	0.039	0.796
6 < *d* ≤ 9	−0.244	0.000	0.066	0.675
9 < *d*	−0.361	0.000	0.015	0.917
Duration since first birth				
1st year	−0.258	0.000	−0.285	0.000
2nd year	Ref.	–	Ref.	–
3rd year	−0.103	0.007	−0.037	0.476
4th year or later	−0.246	0.000	−0.355	0.000
Birth cohort				
1950–1954	−0.005	0.914	0.023	0.721
1955–1959	Ref.	–	Ref.	–
1960–1964	−0.031	0.434	−0.051	0.291
1965–1969	−0.019	0.631	0.005	0.916
Age at migration				
15–19	Ref.	–	Ref.	–
20–24	−0.025	0.489	0.064	0.234
25–29	0.036	0.521	0.050	0.401
30+	−0.053	0.326	0.090	0.312
Education				
Low	Ref.	–	Ref.	–
Intermediate	0.074	0.229	−0.001	0.990
High	0.021	0.817	−0.020	0.739
Other	−0.003	0.936	−0.023	0.691
Marriage and migration history				
Family reunification				
Marriage, R migrated, partner migrated later	0.063	0.436	−0.080	0.267
Marriage, partner migrated, R migrated later	0.043	0.442	0.058	0.767
Marriage migration				
R migrated, marriage + partner migrated	0.078	0.309	−0.024	0.698
Partner migrated, marriage + R migrated	Ref.	–	Ref.	–
Other groups				
Marriage before/at joint couple’s migration	0.006	0.903	−0.025	0.708
R and partner migrated, married later	0.076	0.187	−0.045	0.453
Never married	0.012	0.873	−0.016	0.868
Missing info on marriage or partner’s migration date	0.071	0.137	−0.024	0.699
First child born				
Before migration	Ref.	–	Ref.	–
After migration	0.263	0.000	0.134	0.035
No. of birth events		449		432
AIC		1213.5		979.25

*Notes*: R stands for respondent, a comma means that the event took place after the previous event whereas + means that both events took place around the same time.

*Data*: German GGS 2006, unweighted

## Discussion

The main objective of this paper was to challenge the common understanding that Turkish immigrants who arrived to Germany after 1973 mainly arrived for family reunification, resulting in high fertility during the years immediately following immigration. We used the Turkish sample of the German Generations and Gender Survey and examined timing effects in first and second childbirth among male and female Turkish immigrants in Germany. By taking into account the marriage and migration history of respondents and their partners, we were able to distinguish between family reunifiers, namely those who were married before one of the partners migrated to Germany and who reunified in Germany later on, and marriage migrants. The latter category comprises migrants who immigrated after marrying a partner who was already living in Germany by the time of the wedding. As has been confirmed by our findings, first and second childbirth intensities among Turkish immigrants in Germany vary substantially by duration of stay and between both immigrant groups.

In line with our first hypothesis, evidence for pre-migration disruption of first birth risks has been found only among marriage migrants, but not among those who migrated for family reunification. Even though family reunifiers experience a period of separation before the partner follows his or her spouse to Germany, this does not translate into lower first or second birth intensities in the years before migration. Furthermore, we find differences between marriage migrants and family reunifiers in childbirth timing after immigration to Germany. In line with the hypothesis of an interrelation of marriage, migration and childbirth, the years shortly after arrival in Germany are dominated by high first birth intensities among Turkish marriage migrants. This is more pronounced among women. Furthermore, unlike men, women also experience a similar effect for the second child. However, this arrival effect of high first birth risks is not evident among those who migrated for family reunification. In previous studies, it was argued that high birth intensities immediately after arrival are a typical pattern among family reunifiers, because they are a highly family-oriented group. By contrast, our findings reveal that Turkish migrants arriving to Germany for family reunification neither show elevated fertility shortly after arrival, nor did we find any signs of disruption during the years in which partners were separated. This holds for both male and female respondents. One possible explanation could be that migrants may have used the family reunification channel, as it is one of the few options for legal migration from Turkey to Germany, but that their main reasons for coming to Germany were work-related, so that having children was less important to them at that time. Another explanation could be that family reunifiers already had a child before the spouse followed his or her partner to Germany. This was also shown in our event plots, displaying the age at first childbirth, marriage and migration. As a result, an arrival effect of high birth intensities might be evident for higher parity births only. Regarding our third hypothesis about gender differences in marriage migration, we cannot draw a firm conclusion. On the one hand, it was hypothesized that men who chose a wife from Turkey are particularly traditional, which would be reflected in a high family orientation of these couples and high birth intensities after the immigration of the spouse. On the other hand, women who marry a spouse from Turkey frequently do so not to maintain traditional gender and family roles, but to emancipate themselves from the family-in-law and the traditional family model (Baykara-Krumme and Fuß 2011; González-Ferrer 2006). We find that the arrival effect of high first birth risks is particularly pronounced among female marriage migrants. Women arriving in Germany as marriage migrants thus seem to be a highly family-oriented group. The pattern among men is less clear, but it seems that first childbirth, marriage, and migration are more dispersed among men arriving as marriage migrants compared with female migrants. Interestingly, we did find gender differences regarding the effects of the age at migration. In our sample, men were more likely to have arrived during childhood, while the majority of Turkish women came as young adults. Among women, the age at immigration is a major determinant of first childbirth. Women arriving during young adulthood have particularly high first birth intensities. Even though we could not estimate the interaction effect between age at migration and different marriage and migration histories, this might indicate that especially young women have arrived as marriage migrants who seem to have particularly high first birth rates immediately after immigration. To fully understand gendered patterns in marriage migration from Turkey and the implications for fertility, one would need to compare both male and female migrants within one model and evaluate differences with interaction effects. Because our group of male marriage migrants is quite small and only few of our results are statistically significant, this remains a topic for further research.

Our findings on second childbirth reveal that first birth and migration timing are strong predictors of second childbirth. Having a second child is particularly likely in the second year after the birth of the first child and second birth risks are higher if the first child was born after immigration. Among female Turkish migrants, we find a clear arrival effect indicated by high second birth intensities in the year immediately following migration and decreasing second birth intensities in the following years. In contrast, we did not find any significant effects of individual characteristics such as education, birth cohort or the marriage and migration history on second births. This finding is not surprising given the fact that in Turkey having a second child is quite universal (Yavuz 2008). It would have been helpful to estimate an interaction of the duration of stay in Germany and the time since first birth to enhance the understanding of the relationship between first and second childbirth and migration. Again, for a lack of a sufficiently large sample size, this has to be left to future research.

The question remains in how far Turkish immigrants in Germany are a selective category. It is particularly difficult to distinguish the interrelation of childbirth and migration from selection effects (e.g. Lübke 2014; Milewski 2007). As noted by Lübke (2014), data on the country of origin are needed to disentangle selectivity issues. Since no Turkish data were available, we focused on marital and migration histories. The short time intervals between childbirth and migration we found among marriage migrants does not seem to be a common pattern among Turkish migrants in general, but is apparent for those who came to Germany as a marriage migrant only. This implies that Turkish marriage migrants are a selective group with a strong family orientation. Previously, it was often assumed that immigrants arriving under the family reunification law are highly family-oriented. According to our findings, they may immigrate under the family reunification law, but their fertility behaviour does not reflect a strong family orientation. As a result, the legal channel that is used by immigrants should not be confused with the actual reasons for migration. To better understand fertility patterns and other family-related events, the marital status at migration and the timing of the marriage and the migration of both partners should not be neglected. In addition, our findings are representative of Turkish citizens only, because the GGS sample mainly consists of Turkish immigrants who have lived in Germany for decades and did not move back to Turkey, but do not have German citizenship. However, the share of Turkish immigrants in Germany who naturalized was only 21 % in 2005 (Bandorski et al. 2007). For our comparison of marriage migrants and those who came for family reunification, we took into account the marriage and migration dates of the respondents and their partners. Unfortunately, in the GGS data, only the migration dates of the respondents’ current partner at the time of the interview were surveyed. As a result, our findings are based on a sample of respondents who were still with the same partner as before migration; Since this share is quite high in our sample, this is a relatively minor problem.

Irrespective of these shortcomings, this study adds to the previous literature in several ways. First, it offers detailed findings on male and female Turkish migrants' fertility in Germany. We furthermore show that the high fertility immediately after arrival is not very common among Turkish immigrants arriving for family reunification, but, that it is dominant among marriage migrants. Both marriage migrants and those coming for family reunification experience temporary separation periods in which one partner resides in Germany and the other partner lives in Turkey. But for family reunifiers, this is not reflected in their fertility behaviour. Based on that, we might speculate that marriage migrants and those coming for family reunification have different reasons for migration that result in different first and second birth intensities after arrival. Our results also are of interest for other Western European countries with large Turkish communities such as the Netherlands, France, Austria, Sweden, or Belgium. Evidence for an arrival effect of high birth intensities immediately after immigration furthermore may not only be relevant for other first-generation immigrant groups in Europe, but also for the large community of Turkish migrants’ descendants. In Belgium, for example, the majority of the Turkish second generation chooses a first-generation partner from the country of origin (Landschoot et al. 2014). As a result, marriage migration will probably continue among Turkish immigrants in Europe, possibly accompanied by fertility patterns that are similar to those found in our study. Finally, our findings might motivate to replicate the study for other immigrant communities with high shares of marriage migrants such as, for example Senegalese immigrants in Spain or France or Moroccan immigrants in France, the Netherlands, or Belgium.

## Appendix

**Table 5:** Number of occurrences and exposures for second birth

	Women		Men	
	Person months at risk (%)	Number of second births	Person months at risk (%)	Number of second births
Cohort				
1950–1954	15.5	72	6.8	32
1955–1959	18.0	82	18.3	78
1960–1964	28.5	126	38.5	150
1965–1969	38.1	168	36.4	171
Education				
Low	76.3	333	50.9	215
Intermediate	12.2	63	31.7	147
High	2.3	13	8.7	38
Other	9.1	39	8.6	31
Age at migration				
0–14	27.2	128	37.5	163
15–19	26.4	109	26.3	99
20–24	24.8	117	15.1	81
25–29	7.8	41	11.2	49
30+	13.7	53	9.9	39
Marriage and migration history				
Family reunification				
Marriage, R migrated, partner migrated later	3.8	14	4.7	15
Marriage, partner migrated, R migrated later	10.1	39	0.4	3
Marriage migration				
R migrated, marriage + partner migrated	12.5	53	37.1	157
Partner migrated, marriage + R migrated	22.0	103	8.6	39
Other groups				
Married before/at joint couple’s migration	8.9	43	7.1	31
R and partner migrated, married later	18.6	93	23.4	101
Never married	5.0	13	4.4	14
Missing info on marriage or partner’s migration date	19.1	90	14.3	71
No. of respondents at risk		505		545
No. of birth events		449		432

*Data*: German GGS 2006, unweighted

**Table 6:** Predicted probabilities of having a first birth, interaction effect of the duration of stay and the couple’s marriage and migration history, female Turkish migrants

Combined duration since migration, marriage and migration history	Predicted probabilities	SE
Family reunification		
$d\le -3$, marriage, R migrated, P migrated later	0.404	0.181
$-3 < d \le -1$, marriage, R migrated, P migr. later	0.557	0.179
$-1 < d \le 0$, marriage, R migrated, P migr. later	0.672	0.240
$0 < d \le 1$, marriage, R migrated, P migr. later	0.271	0.234
$1 < d \le 3$, marriage, R migrated, P migr. later	0.276	0.131
$3 < d \le 6$, marriage, R migrated, P migr. later	0.114	0.110
$6 < d \le 9$, marriage, R migrated, P migr. later	0.000	0.000
$9 < d$, marriage, R migrated, P migr. later	0.142	0.100
$d\le -3$, marriage, P migrated, R migr. later	0.476	0.107
$-3 < d \le -1$, marriage, P migrated, R migr. later	0.275	0.120
$-1 < d \le 0$, marriage, P migrated, R migr. later	0.343	0.202
$0 < d \le 1$, marriage, P migrated, R migr. later	0.286	0.132
$1 < d \le 3$, marriage, P migrated, R migr. later	0.060	0.061
$3 < d \le 6$, marriage, P migrated, R migr. later	0.069	0.070
$6 < d \le 9$, marriage, P migrated, R migr. later	0.000	0.000
$9 < d$, marriage, P migrated, R migr. later	0.119	0.086
Marriage migration		
$d\le -3$, R migrated, marriage + P migr.	0.618	0.347
$-3 < d \le -1$, R migrated, marriage + P migr.	0.000	0.000
$-1 < d \le 0$, R migrated, marriage + P migr.	0.000	0.000
$0 < d \le 1$, R migrated, marriage + P migr.	0.000	0.000
$1 < d \le 3$, R migrated, marriage + P migr.	0.593	0.159
$3 < d \le 6$, R migrated, marriage + P migr.	0.236	0.096
$6 < d \le 9$, R migrated, marriage + P migr.	0.341	0.119
$9 < d$, R migrated, marriage + P migr.	0.106	0.052
$d\le -3$, P migrated, marriage + R migr.	0.380	0.105
$-3 < d \le -1$, P migrated, marriage + R migr.	0.243	0.097
$-1 < d \le 0$, P migrated, marriage + R migr.	0.231	0.111
$0 < d \le 1$, P migrated, marriage + R migr.	0.673	0.088
$1 < d \le 3$, P migrated, marriage + R migr.	0.421	0.091
$3 < d \le 6$, P migrated, marriage + R migr.	0.182	0.063
$6 < d \le 9$, P migrated, marriage + R migr.	0.039	0.030
$9 < d$, P migrated, marriage + R migr.	0.047	0.030
Other groups		
$d\le -3$, married before/at joint migration	0.379	0.107
$-3 < d \le -1$, married before/at joint migration	0.119	0.114
$-1 < d \le 0$, married before/at joint migration	0.311	0.160
$0 < d \le 1$, married before/at joint migration	0.470	0.133
$1 < d \le 3$, married before/at joint migration	0.363	0.113
$3 < d \le 6$, married before/at joint migration	0.172	0.082
$6 < d \le 9$, married before/at joint migration	0.187	0.096
$9 < d$, married before/at joint migration	0.037	0.038
$d\le -3$, R and P migrated, married later	0.196	0.129
$-3 < d \le -1$, R and P migrated, married later	0.123	0.119
$-1 < d \le 0$, R and P migrated, married later	0.402	0.219
$0 < d \le 1$, R and P migrated, married later	0.348	0.201
$1 < d \le 3$, R and P migrated, married later	0.641	0.126
$3 < d \le 6$, R and P migrated, married later	0.348	0.096
$6 < d \le 9$, R and P migrated, married later	0.128	0.076
$9 < d$, R and P migrated, married later	0.122	0.055
$d\le -3$, never married	0.521	0.164
$-3 < d \le -1$, never married	0.000	0.000
$-1 < d \le 0$, never married	0.294	0.244
$0 < d \le 1$, never married	0.704	0.226
$1 < d \le 3$, never married	0.207	0.188
$3 < d \le 6$, never married	0.112	0.109
$6 < d \le 9$, never married	0.152	0.145
$9 < d$, never married	0.054	0.055
No. of birth events	310	
AIC	1510.1	

*Notes*: P—partner, R—respondent, Controlled for age at migration, birth cohort, education. *Data*: German GGS 2006, unweighted

**Table 7:** Predicted probabilities of having a first birth, interaction effect of the duration of stay and the couple’s marriage and migration history, male Turkish migrants

Combined duration since migration, marriage and migration history	Predicted probabilities	SE
Family reunification		
$d\le -3$, marriage, R migrated, P migrated later	0.163	0.068
$-3 < d \le -1$, marriage, R migrated, P migr. later	0.119	0.116
$-1 < d \le 0$, marriage, R migrated, P migr. later	0.000	0.000
$0 < d \le 1$, marriage, R migrated, P migr. later	0.215	0.143
$1 < d \le 3$, marriage, R migrated, P migr. later	0.106	0.103
$3 < d \le 6$, marriage, R migrated, P migr. later	0.233	0.107
$6 < d \le 9$, marriage, R migrated, P migr. later	0.177	0.120
$9 < d$, marriage, R migrated, P migr. later	0.000	0.000
$d\le -3$, marriage, P migrated, R migr. later	0.000	0.000
$-3 < d \le -1$, marriage, P migrated, R migr. later	0.499	0.361
$-1 < d \le 0$, marriage, P migrated, R migr. later	0.494	0.362
$0 < d \le 1$, marriage, P migrated, R migr. later	0.286	0.132
$1 < d \le 3$, marriage, P migrated, R migr. later	0.000	0.000
$3 < d \le 6$, marriage, P migrated, R migr. later	0.325	0.276
$6 < d \le 9$, marriage, P migrated, R migr. later	0.000	0.000
$9 < d$, marriage, P migrated, R migr. later	0.119	0.086
Marriage migration		
$d\le -3$, R migrated, marriage + P migr.	0.104	0.063
$-3 < d \le -1$, R migrated, marriage + P migr.	0.000	0.000
$-1 < d \le 0$, R migrated, marriage + P migr.	0.000	0.000
$0 < d \le 1$, R migrated, marriage + P migr.	0.316	0.163
$1 < d \le 3$, R migrated, marriage + P migr.	0.264	0.084
$3 < d \le 6$, R migrated, marriage + P migr.	0.182	0.051
$6 < d \le 9$, R migrated, marriage + P migr.	0.295	0.064
$9 < d$, R migrated, marriage + P migr.	0.135	0.033
$d\le -3$, P migrated, marriage + R migr.	0.108	0.048
$-3 < d \le -1$, P migrated, marriage + R migr.	0.246	0.101
$-1 < d \le 0$, P migrated, marriage + R migr.	0.000	0.000
$0 < d \le 1$, P migrated, marriage + R migr.	0.449	0.123
$1 < d \le 3$, P migrated, marriage + R migr.	0.350	0.091
$3 < d \le 6$, P migrated, marriage + R migr.	0.139	0.061
$6 < d \le 9$, P migrated, marriage + R migr.	0.090	0.054
$9 < d$, P migrated, marriage + R migr.	0.096	0.068
Other groups		
$d\le -3$, married before/at joint migration	0.155	0.060
$-3 < d \le -1$, married before/at joint migration	0.000	0.000
$-1 < d \le 0$, married before/at joint migration	0.310	0.143
$0 < d \le 1$, married before/at joint migration	0.283	0.129
$1 < d \le 3$, married before/at joint migration	0.277	0.101
$3 < d \le 6$, married before/at joint migration	0.165	0.078
$6 < d \le 9$, married before/at joint migration	0.113	0.079
$9 < d$, married before/at joint migration	0.000	0.000
$d\le -3$, R and P migrated, married later	0.097	0.070
$-3 < d \le -1$, R and P migrated, married later	0.000	0.000
$-1 < d \le 0$, R and P migrated, married later	0.000	0.000
$0 < d \le 1$, R and P migrated, married later	0.182	0.123
$1 < d \le 3$, R and P migrated, married later	0.407	0.119
$3 < d \le 6$, R and P migrated, married later	0.227	0.063
$6 < d \le 9$, R and P migrated, married later	0.330	0.076
$9 < d$, R and P migrated, married later	0.091	0.031
$d\le -3$, never married	0.183	0.098
$-3 < d \le -1$, never married	0.000	0.000
$-1 < d \le 0$, never married	0.351	0.286
$0 < d \le 1$, never married	0.389	0.232
$1 < d \le 3$, never married	0.093	0.091
$3 < d \le 6$, never married	0.105	0.101
$6 < d \le 9$, never married	0.250	0.160
$9 < d$, never married	0.104	0.053
No. of birth events	251	
AIC	1186.5	

*Notes*: P—partner, R—respondent, controlled for age at migration, birth cohort, education. *Data*: German GGS 2006, unweighted

## Footnotes

1 International Standard Classification of Education 1997, http://www.unesco.org/education/information/nfsunesco/doc/isced_1997.htm. Unfortunately, no time-varying information on education is available. The variable on education therefore refers to the highest school degree obtained, which can be assumed to be constant over the life course.

2 It has to be noted that only few Turkish immigrants in our sample have married a person who was born in Germany (*n* = 91). This number is too small to form an own category. Therefore, these migrants have been coded into this last category of respondents.
